# Translating Precision Health for Pediatrics: A Scoping Review

**DOI:** 10.3390/children10050897

**Published:** 2023-05-17

**Authors:** Mathushan Subasri, Celine Cressman, Danielle Arje, Leighton Schreyer, Erin Cooper, Komal Patel, Wendy J. Ungar, Melanie Barwick, Avram Denburg, Robin Z. Hayeems

**Affiliations:** 1 Child Health Evaluative Sciences Program, The Hospital for Sick Children Research Institute, Toronto, ON M5G 1X8, Canada; mathushan.subasri@mail.mcgill.ca (M.S.); celine.cressman@sickkids.ca (C.C.); danielle.arje@sickkids.ca (D.A.); leighton.schreyer@mail.utoronto.ca (L.S.); ecoops12@gmail.com (E.C.); kpatel.hba2022@ivey.ca (K.P.); wendy.ungar@sickkids.ca (W.J.U.); melanie.barwick@sickkids.ca (M.B.); avram.denburg@sickkids.ca (A.D.); 2 Department of Paediatrics, University of Toronto, Toronto, ON M5G 1X8, Canada; 3 Institute for Health Policy, Management and Evaluation, University of Toronto, Toronto, ON M5T 3M6, Canada; 4 Division of Haematology/Oncology, Hospital for Sick Children, University of Toronto, Toronto, ON M5G 1X8, Canada

**Keywords:** precision health, pediatrics, evidence development, health technology assessment, priority-setting, implementation, child health policy

## Abstract

Precision health aims to personalize treatment and prevention strategies based on individual genetic differences. While it has significantly improved healthcare for specific patient groups, broader translation faces challenges with evidence development, evidence appraisal, and implementation. These challenges are compounded in child health as existing methods fail to incorporate the physiology and socio-biology unique to childhood. This scoping review synthesizes the existing literature on evidence development, appraisal, prioritization, and implementation of precision child health. PubMed, Scopus, Web of Science, and Embase were searched. The included articles were related to pediatrics, precision health, and the translational pathway. Articles were excluded if they were too narrow in scope. In total, 74 articles identified challenges and solutions for putting pediatric precision health interventions into practice. The literature reinforced the unique attributes of children and their implications for study design and identified major themes for the value assessment of precision health interventions for children, including clinical benefit, cost-effectiveness, stakeholder values and preferences, and ethics and equity. Tackling these identified challenges will require developing international data networks and guidelines, re-thinking methods for value assessment, and broadening stakeholder support for the effective implementation of precision health within healthcare organizations. This research was funded by the SickKids Precision Child Health Catalyst Grant.

## 1. Introduction

Advances in understanding the genetic basis of disease through sequencing technologies are refining diagnostic capabilities, enabling tailored surveillance, offering new therapeutic options, and optimizing treatment delivery. Understanding that different underlying genetic changes can cause the same disease means not all patients with the same problem will benefit from the same treatment. The field of precision health has emerged as part of this paradigm shift, where the integration of genomic technologies into medical management decisions promises to improve the quality of care for specific patient groups, including children [1].

This promise is exemplified in success stories for single-gene disorders such as cystic fibrosis (CF). CF is caused by mutations in the cystic fibrosis transmembrane conductance regulator (CFTR) gene. Through a greater understanding of the molecular pathophysiology of CF, landmark precision therapies have drastically shifted care to treat the underlying cause of the disease [2,3]. One of these therapies is Trikafta, which Health Canada recently approved. This triple combination therapy improves CFTR function for patients with the most common F508del mutation or one of 177 other specified mutations [4]. As a result, most patients in Canada with CF over the age of six can now receive this highly effective therapy to treat the underlying cause of their disease.

Despite these improvements, the challenges in achieving the anticipated impacts of precision health on patient care remain. Given the small study populations and high cost inherent to precision health diagnostics and therapeutics, defining or demonstrating benefit is costly and empirically challenging. This reality often results in high uncertainty, meaning that the adjudication of value is difficult. Other barriers include the lack of long-term safety data on precision therapy side effect profiles and genomically guided patient stratification; insufficient infrastructure to collect, analyze, and store genomic data; and potential ethical implications (e.g., identifying secondary findings or variants of unknown significance). These challenges span the entire translational pathway, from generating clinical evidence, prioritizing resources for regulatory and value assessment services, assessing the clinical and economic value, and ultimately, implementing it into clinical practice [5,6].

The strategies, processes, and frameworks that facilitate translating innovative health technologies from research to clinical practice are plentiful and have evolved from a core focus on clinical safety, effectiveness, and cost to include ethics [7], equity, patient, and public involvement [8], deliberative processes [9], and implementation science. The frameworks that reflect these dimensions are increasingly tailored to precision health [10,11], but the majority do not sufficiently attend to specific populations such as children. As such, current frameworks fail to incorporate the unique physiology and socio-biology of childhood health and illness, compromising their usability for children and potentially jeopardizing the impact that novel discoveries can have on pediatric medicine. While implementation science frameworks are readily applicable to pediatrics, they are not typically integrated into the development stages of research (i.e., hybrid designs) and are only just emerging in precision health. Continuing to resolve these limitations is imperative because children may have the most to gain from precision health interventions through identifying genetic aberrations and tailoring care early in life [12]. The thoughtful integration of the principles and criteria of health technology assessment (HTA) and implementation science in studies throughout the translational pathway is essential for realizing the anticipated impact. To this end, the objective of this study is to synthesize the existing literature on developing evidence, assessing value, and implementing precision health interventions for children.

## 2. Materials and Methods

### 2.1. Definitions

We used scoping review methodology [13,14,15] to identify and describe the criteria and principles used to develop evidence, prioritize, assess value, and implement precision health strategies. Specifically, evidence development refers to the process of reviewing and synthesizing clinical evidence typically generated through clinical trials or real-world evidence data collection; prioritization refers to the process of identifying high-priority interventions for HTA; evidence appraisal refers to the formal HTA process; and implementation refers to the process of translating medical interventions into clinical care. The meaning of “precision health” is evolving and often used interchangeably with precision medicine and personalized, targeted, or individualized health/medicine [11]. For the purposes of this review, we define precision health as preventative, diagnostic, and therapeutic measures that utilize biological information and biomarkers on the level of molecular disease pathways, genomics, transcriptomics, proteomics, epigenomics, and metabolomics. We did not set a definitive age criterion to define pediatrics, as this varies between jurisdictions. Instead, we viewed pediatric medicine as the care provided from birth and throughout childhood and adolescence. 

### 2.2. Search Strategy 

PubMed, Scopus, Web of Science, and Embase databases were selected to ensure comprehensive coverage of journals. Limits were set to exclude non-English articles, those published before the year 2000, and any news items, letters, corrections, retractions, or conference abstracts. The timeframe of January 2000–May 2022 was selected with the rationale that most advancements in and applications of precision medicine occurred after that time, with rapid progress on precision medicine in academic research output and search volume occurring within the last decade. Searches were conducted on 5 November 2022 using keywords focused on pediatrics, precision health, evidence development, priority-setting, evidence appraisal, and implementation (See Appendix A for the complete search strategy). 

### 2.3. Study Selection

Article abstracts were screened by a minimum of two independent reviewers for potential inclusion in the scoping review based on relevance to precision child health. The inclusion criteria were threefold and required articles to be (1) related to pediatrics; (2) focused on precision health; and (3) discussing the translational pathway (i.e., evidence development, prioritization, evaluation, and/or implementation). Abstract screening yielded 134 potentially relevant articles that proceeded to full-text reviews. Full texts were reviewed by at least two independent reviewers, and additional members of the study team were consulted to resolve any uncertainties about relevance. Articles were excluded if they were unrelated to pediatric precision health or had a narrow scope. A narrow scope was defined as focusing only on clinical outcomes, epidemiology, or a single biomarker, test, or therapy without considering implications to evidence development, prioritization, evidence appraisal, or broader clinical implementation. 

### 2.4. Data Extraction and Coding

A data matrix was created to extract and display information from the articles included. The following study characteristics were extracted from each paper: article title, publication year, type of research paper (e.g., original article, review, commentary), jurisdiction (i.e., study setting or, for reviews and commentaries, the country of the senior authors’ primary academic affiliation), and purpose of the article. The research methodology, population and sample size, and disease domain were also documented for papers reporting on primary data collection (henceforth referred to as primary research studies). Additionally, the stages of translational research were used to examine whether the focus of each paper contributed to evidence development, priority-setting, evidence appraisal, implementation, or multiple categories. 

**Figure 1:**
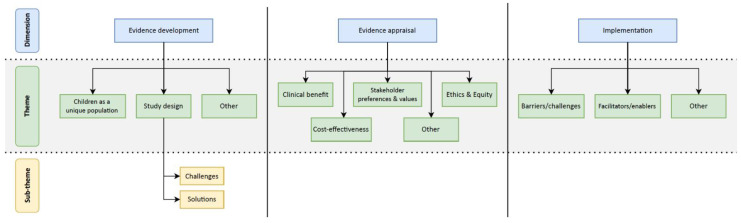
Coding structure used to organize data extraction.

After completing data extraction and full-text review, it became evident that data related to prioritization within the context of precision child health was non-existent. As such, the remaining dimensions—evidence development, evidence appraisal, and implementation—provided an organizational framework to analyze emerging themes related to pediatrics. Within each of these dimensions, the data were coded across several themes (Figure 1). The Ontario Health Technology Advisory Committee (OHTAC) Decision Determinants framework guided the initial identification and conceptualization. OHTAC uses the following five criteria to evaluate new health technologies: the context of the application, benefits and harm, economics, patient-centered care, and feasibility [6]. Anchored by this approach, the codebook was modified and expanded when novel themes were identified, and other themes were revised and refined [16,17]. After coding was completed for all included articles, analytical memos were generated for each dimension. The coded data were reviewed to ensure coherence within a theme, identify patterns and relationships between themes, and highlight any text segments that were particularly illustrative of a theme. Narrative summaries were crafted to capture the key insights for each thematic code and served as guides for further cross-dimensional analysis, which took place through team discussions.

## 3. Results

**Figure 2:**
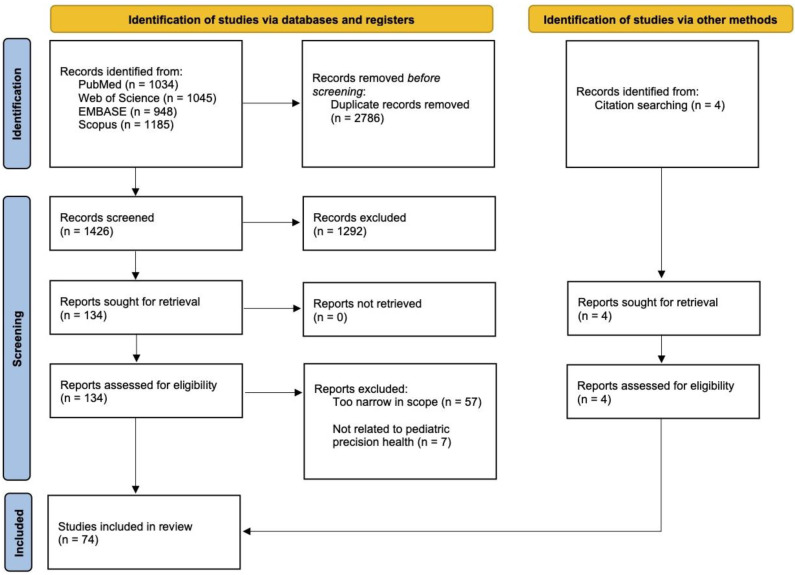
PRISMA flow diagram for the study selection process.

A total of 4212 articles were initially identified, and after filtering to remove duplicates, a total of 1426 articles remained (Figure 2). The abstract screening yielded 134 articles, of which 64 articles were excluded during full-text reviews (Supplementary S1) either because they were unrelated to pediatric precision health (*n* = 7) or were identified as too narrow in scope (*n* = 57). After the full-text reviews were completed, a total of 70 articles remained. A total of 4 additional articles were identified within article reference lists or independently, forming a final set of 74 included articles (Figure 2). 

Of the 74 reviewed articles, the majority were primary research studies (*n* = 28; 38%) and review articles (*n* = 37; 50%). The year of publication ranged from 2003 to 2022, and most were published after 2010 (*n* = 72; 97%). There was broad geographical representation, with 45 papers (61%) published in North America and 29 published (39%) across Europe, Australia, and Asia. Most articles did not have a disease domain of focus; however, of those that did, the most prevalent was oncology (Table 1). 

### 3.1. Thematic Analysis 

Within the evidence development dimension, we identified two major themes which focused on the unique attributes of children and implications for study design. Within the evidence appraisal dimension, we identified four major themes related to assessing the value of various technologies and interventions in the context of precision child health. Finally, within the implementation dimension, the findings largely focused on the challenges and potential solutions for implementing pediatric precision health. No articles within the context of precision child health related to prioritization were identified. The following sections elaborate on these identified themes (Table 2).

### 3.2. Evidence Development

#### 3.2.1. Children Are a Unique Population

A central theme that emerged from the literature related to the ways in which the physiology of children is distinct from adults. In addition to a child’s unique physiology, the molecular events driving disease pathophysiology can significantly differ between children and adults, even within the same disease domain. These differences pose numerous challenges to medical research, including pharmacokinetic safety and pharmacodynamic efficacy. Unfortunately, due to such challenges and reduced profit incentives, many therapies are trialed in and for adults, and then the findings, including how they inform eligibility criteria, are inappropriately extrapolated to children. Additional differences between children and adults related to behavioral and environmental impacts on disease and therapeutic goals [18,19] further complicate the use of adult-trialed therapies in children. Another prominent theme was the notion of increased phenotypic heterogeneity in children compared to adults; namely, children have natural genetic variations that often change throughout their development, impacting gene expression and possibly gene function [18,19,20,21,22,23,24,25,26,27,28,29,30,31,32,33,34]. Moreover, these differences may not only exist between children and adults, but also across the developmental trajectory between infants, children, and adolescents [19,21,22,35]. Specifically, during rapid childhood development, there are age-related differences in drug-metabolizing enzymes and drug receptors. For example, commonly studied cytochrome p450 enzymes (e.g., CYP2D6, CYP2C9) have negligible perinatal activity and only reach adult activity after two weeks of age [36,37]. Ultimately, these differences manifest as differing genotype–phenotype–drug response relationships across child development. For example, a child may not respond to a specific drug dosage extrapolated from adult data because the drug target is not genetically expressed at their specific developmental stage [20]. Strictly using adult data as a proxy for decisions in children jeopardizes the efficacy and safety of precision health interventions.

#### 3.2.2. Study Design Challenges and Potential Solutions

While the unique characteristics of children necessitate child-specific evidence, they also impose distinct challenges to gathering this evidence. The most prominent issue identified in the literature is the rarity of childhood disease and the implications this has for reaching statistical significance in clinical trials [18,19,20,21,23,24,25,26,28,38,39,40,41,42,43,44,45,46,47,48,49,50,51,52,53,54]. The challenge of small sample size is compounded by genotyping, which further subdivides eligible participants into molecular-based strata [41,44,53,54]. As the number of diagnostic or prognostic classifications grows, the reproducibility of findings between one generation of trials and the next becomes increasingly challenging [54,55]. The reproducibility is further threatened by the higher degree of heterogeneity within the pediatric patient population [20,23], the difficulties in assessing endpoints in pediatric clinical trials as ailments are carried into adulthood [19,21,27,40], and the inability to develop a consensus on methodological guidelines or clinical trial protocols due to the rapid growth of precision medicine research and the variable availability of treatment options [19,20,26,28,35,39,41,50,53,56,57,58]. 

Beyond the challenges within clinical trials, there are barriers to initiating trials from both ethical and financial perspectives. Ethically, there is a “historical reticence to involve children in clinical research” [51] due to their inherent vulnerability, the potential inability to provide informed consent, and safety concerns from negative impact during critical periods of development [18,21,23]. For example, performing invasive procedures to collect samples for testing is often not acceptable in children unless there is a clear clinical indication [18,42,59,60]. One solution is to shift toward non-invasive sample collection techniques, such as using saliva for genomic testing [59,61]. Understandably, specific ethical considerations and protections are in place that restrict the risk to which children can be exposed during clinical trials [18,24,25,28,49,51]. Financially, there is limited funding and interest from pharmaceutical companies because of the high costs, time-consuming nature of trials, and small patient populations to engage in pediatric precision health research [19,27,52,62]. 

Considering that generating child-specific evidence is the first step in the health technology development pathway, robust solutions are required to address this bottleneck. Novel clinical trial designs, research collaboration, and standardized guidelines are the main ideas proposed in the literature to tackle small sample sizes. First, several papers suggest moving away from traditional phase III randomized clinical trials and toward designs such as basket trials, umbrella trials, or n-of-1 trials that accommodate for the uncertainty of targetable genetic alterations and treatment availability [49,51,53,54]. 

Second, building collaborative partnerships to recruit patients and share clinical data is seen as essential to adequately power studies [19,24,25,26,28,42,43,48,50,53]. The idea of biobanking samples to build a library of genomic information was well received by patients, families, healthcare providers, and research investigators [24,42,63] and could serve as a shared resource. Many cited the need for collaboration across all levels of stakeholders—from patients and physicians to hospital administrators and government regulators [19,26,48,50,53]. 

Third, standardized guidelines should be developed to facilitate collaboration to maintain research quality and integrity and enable the reproducibility of findings [25,28,33,51]. The guidelines should be evidence-based and address the essential components of evidence development, including methodological and analytical approaches and ethical considerations. For example, the factors identified for the successful recruitment of pediatric patients in trials included the presence of an experienced study coordinator, integration of the study with clinical care, fostering an ongoing team–patient relationship, and having an overlap between the research staff and clinical teams [64]. Increasing the quantity and quality of precision health evidence in children will also require governmental initiatives to compel or incentivize pharmaceutical companies to conduct equivalent studies in children when applicable [18,49,52] and develop the appropriate infrastructure to conduct large-scale collaborative clinical trials [24].

### 3.3. Evidence Appraisal

#### 3.3.1. Clinical Benefit

There was a broad consensus in the reviewed literature that precision health offers potential clinical benefits to patient care. The clinical benefits include accurate diagnoses, reduced treatment side effects, and informed predictions related to early response or relapse. While there is an agreement that the efficacy and safety of precision strategies must be evidence-based, some disease domains, such as oncology, have accumulated more evidence to date. Other domains, such as psychiatry, still lack sufficient evidence [25,27,65,66]. Much of the available evidence was generated in and for adults, yet there are clear indications for precision health interventions in the pediatric setting [31,67]. 

Pediatric precision health is unique because of future health implications for children themselves and their immediate family members. The literature reflects a greater emphasis on pathogenic germline mutations in pediatric cancers, meaning parents and siblings could also benefit from the knowledge generated through the child’s genomic testing [49]. This benefit is not limited to oncology but includes any disease domain with a large genetic component that follows Mendelian inheritance. Moreover, genomic testing early in life, through programs such as newborn screening, can provide the opportunity for the prevention of or reduction in disease progression [24,47,49,58,68]. Moving forward, large-scale prospective genomic studies, especially wherein the complete genome is interpreted rather than individual genes [69], will help identify novel targets and shape which interventions should be prioritized for trials or funding [19,24,39,42,43,52,65]. Since the development of therapeutic agents is driven by only a small fraction of diseases [24], the overall clinical utility of these tools relies on the population and not on the individual level [70]. As this field evolves, other “omic” patterns, such as epigenomics, are emerging as essential contributors to effective clinical evaluation [55], which remains inclusive of the patient’s environment and lifestyle [33,70].

#### 3.3.2. Cost-Effectiveness 

Cost-effectiveness was identified as a vital element of evidence appraisal in the pediatric precision health literature. Most discussions about cost-effectiveness focused on pharmacogenomic testing and the potential health system savings from preventing adverse drug reactions. Specifically, the concept of preemptive testing (i.e., before initiating therapy), as opposed to reactive testing (i.e., after an adverse drug reaction), was argued to be cost-effective as it can reduce the burden on the health system. Evidence of cost-effective preemptive testing was demonstrated for some gene–drug combinations [22,71]. However, the evidence for preemptive testing is less robust in children [67]. Several authors noted that assuming sequencing costs decline, genome-wide sequencing strategies will become more cost-effective than targeted gene testing [49,72]. However, others noted that additional costs warrant inclusion in economic evaluations, such as costs of alternative treatments, extra monitoring, and costs related to implementing and delivering a comprehensive precision health program (e.g., computational power and data storage, necessary personnel) [19,49,73]. The inherent high cost of precision therapies was viewed as a significant barrier to access. If precision therapies are shown to be cost-effective, provider acceptance and adoption are expected to improve, and governments and insurance providers would be more likely to support the implementation [25].

#### 3.3.3. Stakeholder Preferences and Values

HTA typically incorporates the values and preferences of various stakeholders, including patients, healthcare providers, and disease domain experts. Parents’ values and preferences about precision health centere around clinical benefit as it relates to survival, quality of life, and accuracy of testing. Other parental values and preferences involving the administration of precision interventions include honesty about the impact on the clinical course; risks of using novel therapies and managing expectations; the turnaround time for test results; privacy; shared decision making; convenience; cost; human elements of care; and social justice [25,61,74,75]. Generally, patients and families view precision medicine as an opportunity to find answers to help their children and the rest of their family, and to provide future benefits for other children [24,38,42,62]. In one study, adolescents revealed altruistic motivations and hoped that their participation would mean others would not need to experience the same struggles [76]. Precision therapies that are available for specific diseases are often only accessible as a last-line option. As such, it is understandable why some patients and families had low expectations for the clinical impact, felt they had no real choice about whether to pursue a precision therapy, and relied heavily on their child’s healthcare provider for making medical decisions [61,76]. 

The literature suggests that the healthcare providers were also generally accepting and enthusiastic toward precision health interventions [32,34,59,67] and reported that the quality of scientific evidence, clinical benefit to the child, family preferences, hope for children where no hope existed previously, and their sense of satisfaction with the care were all important in their decision making to utilize precision health interventions [61,62,77]. Despite this enthusiasm, there were also some opposing views about the application of pharmacogenomic testing. Some providers reported feeling an ethical obligation to consider precision testing if available, but the obligation to offer precision testing extends only when standard treatments are non-responsive, when there are elicit adverse drug reactions, or when it is recommended by a regulatory-approved guideline [66]. In contrast, other providers believed that precision testing should be performed preemptively as often as possible [71]. 

Two studies gathered the perspectives of a range of stakeholders, including those involved in policy (i.e., development, regulation, and evaluation) and those involved in the application or use of precision child health interventions (i.e., clinicians, parents, and patients) [51,78]. Despite the breadth of perspectives represented, there was an agreement that critical gaps exist in the current HTA process and structure. These stakeholders agreed that the current system is tailored for adults and that utilizing current frameworks for evaluating precision health interventions for children is insufficient. Second, they agreed that a novel child-specific framework is required to provide structure to the unique value proposition for children, but also provide flexibility, given the context of developing efficacy and safety evidence in the evolving precision health space. Such a framework should consider the implications on a child’s future life course; the unmet need in the disease domain; the value of hope; the family impacts; and the voices of children, child health experts, family and caregivers, and the general public [51,78]. The other considerations that were deemed important in economic evaluations for children were age suitability, recall period for impacts on health-related quality of life, and responsiveness across disease courses [79].

#### 3.3.4. Ethics and Equity in Value Assessments 

The ethical challenges of precision child health were described in the following three distinct contexts: challenges encountered in recruiting children into research studies (in “Study Design Challenges and Potential Solutions”); challenges with integrating genomic findings into clinical decisions (in “Implementation Barriers & Enablers”); and challenges for value assessments. These challenges can be divided into substantive and procedural ethical issues. Substantive issues include challenges related to the content and outcomes of decisions, whereas procedural issues relate to the process by which decisions are generated. 

One key substantive issue that was identified was ensuring that precision health interventions for children are assessed fairly, that is, within the context of the unique physiology and socio-biology of childhood health and illness [51,78]. Methodologically, there is potential for penalizing the quality-adjusted life years (QALYs) gained in children if the life course is not adequately accounted for (i.e., if adults are modelled the same as children). In addition, the evidentiary challenges present in the studies conducted on children, as outlined above in the evidence development section, further creates an environment to yield negative assessments [80]. Additionally, the clinical benefit of high-cost interventions may be unfairly reduced in child assessments since health benefits may only be realized later in life [80]. 

A prominent procedural issue surrounds the incorporation of children’s voices into decision making. The perspectives of patients and the general public are identified as increasingly valuable for HTA; however, those of pediatric patients remain under-incorporated. Determining the substantive appropriateness of pediatric perspectives is another challenge that requires a suitable process to be in place and one that is missing from current value assessment procedures [40]. 

Finally, from an equity standpoint, the primary concerns were related to either the cost of delivering interventions or patient health literacy, particularly related to creating and exacerbating potential inequities in access [46,61,66,73]. Concerns related to the social determinants of health should be considered in value assessments because funding for interventions that are not widely accessible will only exacerbate existing health disparities.

### 3.4. Implementation 

#### Barriers and Enablers to Use

While clinical benefit and cost-effectiveness considerations are paramount, the feasibility of implementing precision health interventions is also critical to their effectiveness. For children to safely experience the benefits of precision health, the following two dimensions of implementation were described in the included studies and warrant consideration: (1) providers’ confidence to prescribe and use these interventions and (2) ensuring patients can make informed decisions regarding their care. 

The challenges related to the health system and the provider were (1) gaps in provider comfort; (2) gaps in clinical evidence; and (3) gaps in the system capacity. A key barrier to adopting precision health interventions was clinicians’ lack of comfort with precision health (e.g., interpretation of findings, treatment selection, and awareness of available resources) [20,22,24,38,59,61,65,66,77,81,82,83]. The uncertain clinical significance of some findings and the limited guidelines related to the therapy selection [21,22,34,49,50,51,52,53,57,66,69,76,79,81,84] pose additional challenges for providers seeking to integrate genomic information into their care plans. Proposed solutions to tackling this issue involve developing practice guidelines, building collaborative partnerships, and instituting better educational strategies, including the incorporation of precision health topics into medical school curricula [21,65,66]. 

A second notable barrier, magnified in pediatrics, is the lack of evidence on precision technologies’ safety and long-term effects. This paucity of evidence alters the risk–reward ratio for providers and shifts provider use of precision health interventions toward those in more dire need [50]. To address the uncertainty of evidence, a strong relationship between the provider and the genome analyst is critical to help both stakeholders work synergistically to develop an ideal therapeutic plan [75]. Additionally, incorporating genomics expertise into clinical teams, such as creating a molecular tumor board, can mitigate the challenge of interpreting and communicating findings to patients [30,58,77]. Communicating clinically relevant information between the multidisciplinary clinical team is an asset to healthcare providers’ professional development and clinical decision making [24,28,77]. An additional solution to help providers effectively communicate genomic findings is integrating genomic information into electronic health records (EHRs) and including mechanisms to automatically alert providers to potentially clinically relevant genomic variants [50,52,66,81,83,85]. 

A third barrier to implementing precision health interventions is the health system’s limited capacity to provide access to these interventions. Multiple studies demonstrated that the lack of available genotype-driven treatments serves as a significant barrier for providers [39,41,44,73,86]. Generating additional pathways to access therapies within the health system can catalyze provider uptake of precision interventions. Specific barriers to implementation may vary depending on an institution’s experience and the volume of precision interventions. For example, low-use sites may struggle with personnel and interpreting genomic findings, while high-use sites may struggle with incorporating findings into EHRs and minimizing costs [81]. The feasibility of using genomics to inform clinical decisions is limited by the time and personnel required to process samples, analyze and store data, and interpret findings [24,29,84,87,88]. However, certain institutions have shown that analyzing patient samples and making timely clinical decisions is feasible [44]. Implementing the necessary enablers for success will rely heavily on institutional champions who advocate for precision child health interventions [43,88]. Ultimately, effective prescriptions of precision health interventions for children will require healthcare institutions, providers, and scientists to work with governmental agencies such as regulatory and HTA bodies [21,40]. The rapid pace of innovation in precision health necessitates frequent HTA re-evaluation, posing an additional burden on evaluators and funders [65]. To ensure that patients receive the optimal evidence-based interventions, there is a need for real-world evidence that incorporates data from patient registries, both from industry data and from academic and/or clinical data [51]. 

Finally, the provider–patient relationship presents challenges to the implementation of precision health. The challenges include patient education and consent, the dissemination of genomic findings, and the implications of findings for patients and their families. Pre-intervention counselling can be challenging for providers; however, it is vital, and parents want to be educated on the intervention’s risks, benefits, turn-around time, implications, and accuracy [75]. Effective counselling should avoid the use of medical jargon [22,75,86], be delivered across multiple meetings to avoid overwhelming patients [65,75], and involve research staff when clinical trials are involved [27]. Many authors suggest that during the counselling step, it is imperative that providers and researchers manage parental expectations and thoughtfully communicate the potential and anticipated clinical efficacy and safety [22,25,27,43,59,67,73,77]. Another prominent challenge to implementing these interventions is the patient consent process, as there are varied opinions on the involvement of children [18,25,27,58,88,89]. Those who argue that children should not be involved maintain that children may feel intimidated and that some parents do not want their children present at the initial diagnosis meeting [63,75]. Others argue that involving children improves trust and the success of clinical research and suggest that when children provide assent, it should be personalized to each patient to respect their developing autonomy and capacity [63]. When engaging in the consent or assent process, it is recommended that providers use validated tools and mediums that focus on concrete steps such as blood drawing before addressing abstract topics such as genomic data and privacy [63]. 

Once the intervention has been administered, there are challenges that involve post-intervention counselling. With respect to genomic profiling, some tests are focused on a specific aberrant gene, while others decipher larger portions of the patient’s genome. While more extensive genomic studies can be beneficial, they can potentially identify other clinically relevant molecular aberrations beyond the scope of the patient’s presenting complaint, called secondary findings [27]. Some families accept the disclosure of secondary findings when significant preventative action can be taken; however, the uncertainty of the impact of many genomic variants can also cause anxiety and psychological distress to families [58,65,89]. To address this challenge, it is essential to have recurring discussions on this potential outcome and to use published guidelines to determine which secondary findings require disclosure. The discussions on secondary findings can be complex and stressful; thus, it is recommended to have pre- and post-intervention psychological support [58,65,75]. Some institutions implemented parent boards to continue dialogue surrounding these ethical challenges when working with children and precision health interventions [24,57]. Due to the ability for patient samples and data to be reused, post-intervention counselling is not limited to immediate follow-up. Parents have expressed concerns regarding their personal data’s privacy and believe that institutional and federal protections should be put in place [25,27,42].

## 4. Discussion

This scoping review summarizes the existing literature on the evidence development, appraisal, and implementation of precision health strategies for children, identifying key challenges, potential solutions, and fair criteria to guide value assessments. The predominant finding across the literature reviewed is that children and precision health each present unique challenges along the research and translation pathway, and combining the two poses additional complexity. Precision health for children is a novel and rapidly developing sphere of medicine and research, and this scoping review is the first to synthesize the often-siloed topics of evidence development, evidence appraisal, and implementation. 

Within the evidence development category, the literature reinforced that children are not simply “small adults”; rather, their underlying pharmacokinetics and disease-driving genetics may differ from adults for the same disease. As such, rather than routine extrapolation of adult data to children, careful thought should be given to the appropriate circumstances and parameters for extrapolation, and emphasis should be given to the generation of pediatric data to inform the development of precision health interventions for children. Generating this data is not without its challenges, namely, small sample sizes and unique ethical considerations. Building collaborative multi-institutional networks to standardize protocols and reinforce data sharing are tangible action items. Once sufficient data have been collected, health systems often require novel interventions to undergo HTAs. Some HTA agencies have introduced methods to determine how to prioritize submissions for review [90,91]. Appreciating the pace of precision health research, rapid and frequent reviews will be vital to facilitate access to state-of-the-art interventions. This means methods to prioritize precision health submissions will be important. Interestingly, no papers addressing the prioritization for HTA for precision child health were identified in the reviewed literature. 

The values and preferences for guiding the appraisal of precision child health mirrored those conventionally used in HTA, including cost-effectiveness, level of evidence for clinical benefits and risk, potential impact, and ethical administration. Precision child health as a novel discipline was generally accepted by patients, families, and providers, with the major point of contention being the limited and uncertain evidence upon which to assess value. In an environment of uncertain evidence, interventions are often only available as a last resort after failing other lines of treatment or through participation in clinical trials research. Without accommodating for the challenges in evidence development, few precision child health interventions will be prioritized or receive a positive funding recommendation within existing adjudication frameworks. Therefore, equitable reviews will require changes to both the substantive content and procedures of current HTAs to accommodate the needs of children. Substantively, applying a pediatric lens to uncertain evidence, for example, by considering life course and impacts on developmental trajectories rather than more immediate clinical outcomes—including criteria such as harm reduction and hope—and considering the future benefit to genomics research, could contribute to more favorable assessments of precision interventions [80]. Procedurally, the incorporation of child voices is paramount to decisions that ultimately impact their health and wellness. In clinical settings, health regulatory bodies have defined procedures for gathering informed consent from children for the purposes of making treatment decisions or for recruitment into research studies [92,93,94]. Other non-medical domains of practice where a child’s health and wellness are of interest, such as family law, have developed explicit procedures for incorporating and assessing the level of influence a child’s perspective should have on court proceedings [95]. For example, the Voice of the Child Report is a structured and impartial procedure adopted across Canada for doing so and is subsequently evaluated by judges using a set of eleven criteria. Thus, procedures and explicit criteria need to be developed to systematically incorporate the child’s voice into value assessments [95]. Within health systems research, it is encouraging to see that efforts are underway to recognize the need to build an HTA framework that captures the distinct needs of children more comprehensively and incorporates the child’s voice in these discussions [96]. 

Post-appraisal, key infrastructural changes are required to implement precision health interventions for children. As it stands, the health system is not equipped to realize the potential of ongoing precision health research. For example, improved provider awareness and education are vital for the efficacious prescription of interventions and advocacy for institutional investment in key resources, such as genetic analysts, to guide interpretations of results, genetic counsellors to manage the complex and dynamic nature of genetic research and medicine, and digital technology to handle the volume and complexity of genetic data. Successful implementation will also hinge upon improving pathways to access interventions when indicated. Safe and equitable access should be paramount, especially since precision medicine has the potential to increase health disparities [12]. 

A range of ethical challenges, including a number particular to pediatrics, were seen across evidence development, evidence appraisal, and implementation. Most notably, these challenges include the ethical considerations of clinical trials in children, the ethics of fairness in HTA for pediatric interventions, and the ethical implications of extracting, managing, and disseminating pediatric genetic information. Taken together, these challenges highlight how children are a unique population with distinct needs and inherent vulnerability. Unfortunately, as it stands, the translational research pipeline is insufficient for the needs of children—evidence of this is seen through the lack of pediatric indications and the reliance on off-label or compassionate use of therapeutics in pediatrics [97], as well as limited policy and regulation from national or international health, genomics, and health technology assessment agencies. For example, the FDA provides some regulatory oversight of precision health and children independently, but does not explicitly mention children within precision health [98]. Similarly, the National Institute for Health and Care Excellence (NICE) recently released an updated evaluation manual where they acknowledge there are circumstances where evidence generation is difficult (i.e., rare diseases, children, innovative and complex technologies) and in such cases, a higher threshold of uncertainty may be accepted; however, there are no defined tools or criteria for doing so that are published [99]. 

In general, the dominant framing of precision child health amongst these agencies was one of anticipation for future interest, signaling awareness of this area as in need of attention but offering little guidance or action at present. The highlighted challenges from these agencies are similar to the findings from this scoping review, including low levels of data availability, a lack of industry interest due to lower profitability, a lack of provider knowledge regarding precision health, and few to no clear guidelines for implementation. Reports from the Australian Pharmaceutical Benefits Advisory Committee, Canadian Pediatric Society, Genome Canada, and other bodies have highlighted the need for a review of the HTA system to refine processes and methods of evaluation for new genomic and precision medicine technologies, especially those with clinical applicability to children [100,101,102]. Ultimately, the broad uptake of precision health interventions for children will require upstream prioritization of funding and regulations. For example, Genome UK emphasized the need to increase funding for Genomic Medicine Services to fast-track diagnosis for critically ill pediatric patients with rare genetic diseases [103]. Examples of initial action in this direction were demonstrated through “pediatric rule” documents such as the FDA Research to Accelerate Cures and Equity (RACE) Act and the Pediatric Regulation outlined by the European Medicines Agency (EMA). These two pieces of landmark legislation outline that any applications for rare and orphan drugs and biologics should include assessments of pediatric use to prevent the off-label or inaccessible use of these drugs and technologies in children [104,105]. The early success of these legislations and other regulations that enforce and incentivize drug development for pediatric diseases, especially for rare and orphan diseases, has started to show an increased trend of approved pediatric indications [97,106]. Enacting such legislation may be warranted in other regions as a method to help shift care for pediatric patients with rare diseases away from off-label prescriptions. 

The challenges with structured value assessments and downstream implementation remain largely unaddressed for precision child health. A review of HTAs for gene therapies across various agencies found discrepancies between their economic evaluations due to the lack of a generalized framework for gene therapies [107]. While this is currently being overcome by adjusting frameworks to accommodate for circumstances where evidence generation is challenging, the unsystematic methodology creates barriers to public access. For example, based on current frameworks in Canada, many drugs for rare diseases receive a “list with condition” and price reduction recommendation from CADTH [108]. Delays between each step along this approval and funding pathway take the opportunity away from patients to access vital therapies. A better alignment between agencies could significantly reduce the time children wait to access precision health interventions [35]. One suggested solution not specific to pediatrics but relevant to precision health is using life-cycle health technology assessment (LC-HTA) [109,110]. Under LC-HTA, the intervention is iteratively appraised throughout the translational pipeline and relies on managed access and real-world evidence to guide further investment or disinvestment and ultimately expedite public access. Nonetheless, any value assessment, life-cycle or not, will require explicit criteria to navigate the uncertainty and dynamic nature of precision child health. 

One limitation is that our scoping review did not capture all spheres of precision health, notably, screening and surveillance interventions. For example, wearable digital devices or machine learning algorithms that calculate the optimal screening intervals for chronic metabolic illnesses or cancer syndromes were not captured. Diagnostics may also not have been wholly captured, as our search findings were primarily related to pharmacogenomics and precision therapeutics. Diagnostic tests for children are typically administered when the clinical context suggests an inherited or congenital disease. While the aspects of delivery and use of precision strategies were highlighted, implementation science per se was captured in limited ways. It is important to note that the challenges for evidence development, evidence appraisal, and implementation may manifest differently for different types of precision health interventions. Terminology is also important, and the scope of “precision health” and its variable and evolving interpretations may not capture all the literature that focuses on “omic” guided medical decisions [12,111]. Finally, to appreciate the precision child health literature as a whole, we did not limit our scoping review to the primary literature. Thus, the frequency of specific challenges may be under- or over-reported relative to one another. Nonetheless, the challenges outlined by this scoping review for translating precision child health are valid and require attention to achieve their anticipated impact.

## 5. Conclusions

Precision health interventions have already improved the quality of care for specific patient groups, and the field continues to grow into specialties such as psychiatry and infectious disease [112,113]. Translating this growth into clinical practice in an equitable and sustainable manner is the next challenge. The unique attributes of child health and illness have significant implications on the translational medicine pathway. The inherent challenges with generating evidence for pediatric interventions and the nuances of pediatric cost–benefit need to be better accounted for by current value assessment models and methods. These barriers are exacerbated in the context of precision health, in addition to the novel implementational challenges and ethical and equity concerns surrounding “omic” interventions. Further, when policies and procedures are in place for safe and efficacious utilization, providers and patients perceive precision child health positively. As more attention is brought to precision child health, we need to (1) develop health technology frameworks and principles for value assessment that attend to the unique needs of children and the distinct context of precision medicine; (2) integrate precision child health principles throughout the research translation process; and (3) encourage cooperation between agencies and key stakeholders to ensure that these checkpoints do not serve as a barrier to access.

## Figures and Tables

**Table 1:** Characteristics of articles included in the scoping review (*n* = 74).

	Number	Percentage
**Year of publication**		
Before 2010	2	2.7
2010–2015	10	13.5
2015–2020	24	32.4
2020–2022	38	51.4
**Type of article**		
Review article	37	50
Primary literature	28	37.8
Book chapter	3	4.1
Opinion piece	2	2.7
Editorial	1	1.35
Conference proceedings	1	1.35
Best practice guidelines	1	1.35
Special Report	1	1.35
**Jurisdiction**		
United States of America	33	44.6
Multinational	10	13.5
Canada	12	16.2
Australia	4	5.4
The Netherlands	4	5.4
United Kingdom	3	4.1
Denmark	2	2.7
Israel	1	1.35
Lebanon	1	1.35
Germany	1	1.35
China	1	1.35
Belgium	1	1.35
Spain	1	1.35
**Medical discipline** (if applicable)		
Oncology	29	76.3
Psychiatry	4	10.5
Pulmonology	2	5.3
Rare Diseases	1	2.6
Infectious Disease	1	2.6
Multidisciplinary	1	2.6

**Table 2:** Overview of themes identified.

Dimension	Theme	Description
Evidence Development	Children are a unique population	Children have a unique physiology that is distinct from adults and that changes with age and development. Developmental progression can have large impacts on gene expression and function, ultimately impacting the efficacy of targeted interventions.
	Study design challenges and potential solutions	Small sample sizes, diversity of study protocols, and safety are all barriers to conducting effective clinical trials in the pediatric population. Employing novel trial designs (e.g., umbrella, basket, or n-of-1 trials) and institutional collaboration are potential avenues to circumvent major study design challenges.
Evidence Appraisal	Clinical benefit	As in adult populations, disease domains with sufficient evidence have clear indications for precision health interventions in children. Unique to pediatrics are the potentially greater life course implications and impact on family members through a greater prevalence of germline mutations in cancer predisposition genes.
	Cost-effectiveness	Similar to value assessments for adult interventions, cost-effectiveness was deemed important. Specifically, preemptive pharmacogenomic testing was highlighted as a strategy for health system savings.
	Stakeholder preferences and values	HTA typically incorporates the values and preferences of various stakeholders, including patients, health care providers, and disease domain experts. Parents and providers were generally accepting of precision health interventions. Parents viewed the intervention as an opportunity to improve care but also felt little choice as their children often had advanced stages of disease. Key decision-makers were critical of existing tools and frameworks to evaluate precision health for children.
	Ethics and equity	Substantive and procedural ethical challenges were identified, which included metrics for measuring value for children and the process of incorporating child voices into value assessments, respectively. Equity concerns related to the cost of precision health interventions were raised, as were concerns related to precision health literacy.
Implementation	Implementation barriers and enablers	Challenges with the implementation of precision health interventions relate to (1) providers’ confidence to prescribe and use these interventions, and (2) ensuring patients can make informed decisions regarding the use of these interventions in their care.

## Data Availability

The data and processes presented in this study are available in the Appendix A and Supplementary Material.

## Supplementary Materials

The following supporting information can be downloaded at https://www.mdpi.com/article/10.3390/children10050897/s1, Supplementary S1: Articles excluded after full-text review; Supplementary S2: Characteristics of articles included in the scoping review.

## Appendix A. Search Strategy

PubMed Search (5 November 2022): ((pediatric[Title] OR paediatric[Title] OR child*[Title] OR adolescen*[Title] OR youth[Title] OR young[Title] OR teen*[Title] OR juvenile[Title]) AND (Personalized[Title] OR Personalised[Title] OR Precision[Title] OR Stratified[Title] OR Individuali*[Title] OR Tailor*[Title] OR Pharmacogen*[Title] OR “Health Technolog*”[Title])) AND (priori*[Title/Abstract] OR assess*[Title/Abstract] OR evaluat*[Title/Abstract] OR implement*[Title/Abstract] OR framework*[Title/Abstract] OR guideline*[Title/Abstract]) Timespan: 2000–2022 Language: English Article Type: Exclude “Corrected and Republished Article”, “Letter”, “News”, “Newspaper Article”, “Retracted Publication”, “Retraction of Publication” Web of Science Search (5 November 2022): (TI=(pediatric OR paediatric OR child* OR adolescen* OR youth OR young OR teen* OR juvenile) AND TI=(Personalized OR Personalised OR Precision OR Stratified OR Individuali* OR Tailor* OR Pharmacogen* OR ”Health Technolog*”) AND ((TI=(priori* OR assess* OR evaluat* OR implement* OR framework* OR guideline*) OR (AB=(priori* OR assess* OR evaluat* OR implement* OR framework* OR guideline*))))) Timespan: 2000–2022 Document Types: Exclude: Meeting Abstracts, Letter, Correction Language: English Embase Search (5 November 2022): 1. ((pediatric or paediatric or child* or adolescen* or youth or young or teen* or juvenile) and (Personalized or Personalised or Precision or Stratified or Individuali* or Tailor* or Pharmacogen* or “Health Technolog*”)).ti. and (priori* or assess* or evaluat* or implement* or framework* or guideline*).ab. 2. limit 1 to (English language and yr=“2000-Current”) 3. limit 2 to (article or article in press or books or chapter or conference paper or “conference review” or editorial or “review” or short survey) Scopus Search (5 November 2022): TITLE (pediatric OR paediatric OR child* OR adolescen* OR youth OR young OR teen* OR juvenile) AND TITLE (personalized OR personalised OR precision OR stratified OR individuali* OR tailor* OR pharmacogen* OR “Health Technolog*”) AND TITLE-ABS (priori* OR assess* OR evaluat* OR implement* OR framework* OR guideline*) AND (EXCLUDE (DOCTYPE, “no”) OR EXCLUDE ( DOCTYPE, “le”) OR EXCLUDE (DOCTYPE, “er”) OR EXCLUDE (DOCTYPE, “tb”) OR EXCLUDE (DOCTYPE, “Undefined”)) AND (LIMIT-TO (PUBYEAR, 2022) OR (LIMIT-TO (PUBYEAR, 2021) OR LIMIT-TO (PUBYEAR, 2020) OR LIMIT-TO (PUBYEAR, 2019) OR LIMIT-TO (PUBYEAR, 2018) OR LIMIT-TO (PUBYEAR, 2017) OR LIMIT-TO (PUBYEAR, 2016) OR LIMIT-TO (PUBYEAR, 2015) OR LIMIT-TO (PUBYEAR, 2014) OR LIMIT-TO (PUBYEAR, 2013) OR LIMIT-TO (PUBYEAR, 2012) OR LIMIT-TO (PUBYEAR, 2011) OR LIMIT-TO (PUBYEAR, 2010) OR LIMIT-TO (PUBYEAR, 2009) OR LIMIT-TO (PUBYEAR, 2008) OR LIMIT-TO (PUBYEAR, 2007) OR LIMIT-TO (PUBYEAR, 2006) OR LIMIT-TO (PUBYEAR, 2005) OR LIMIT-TO (PUBYEAR, 2004) OR LIMIT-TO (PUBYEAR, 2003) OR LIMIT-TO (PUBYEAR, 2002) OR LIMIT-TO (PUBYEAR, 2001) OR LIMIT-TO (PUBYEAR, 2000)) AND (LIMIT-TO (LANGUAGE, “English”))
